# Acute and chronic nephrotoxicity of platinum nanoparticles in mice

**DOI:** 10.1186/1556-276X-8-395

**Published:** 2013-09-23

**Authors:** Yoshiaki Yamagishi, Akihiro Watari, Yuya Hayata, Xiangru Li, Masuo Kondoh, Yasuo Yoshioka, Yasuo Tsutsumi, Kiyohito Yagi

**Affiliations:** 1 Laboratories of Bio-Functional Molecular Chemistry, Graduate School of Pharmaceutical Sciences, Osaka University, Suita, Osaka 565-0871, Japan; 2 Laboratories of Toxicology and Safety Science, Graduate School of Pharmaceutical Sciences, Osaka University, Suita, Osaka 565-0871, Japan

**Keywords:** Nanosized materials, Platinum particles, Kidney, Nephrotoxicity, Safety evaluation

## Abstract

Platinum nanoparticles are being utilized in various industrial applications, including in catalysis, cosmetics, and dietary supplements. Although reducing the size of the nanoparticles improves the physicochemical properties and provides useful performance characteristics, the safety of the material remains a major concern. The aim of the present study was to evaluate the biological effects of platinum particles less than 1 nm in size (snPt1). In mice administered with a single intravenous dose of snPt1, histological analysis revealed necrosis of tubular epithelial cells and urinary casts in the kidney, without obvious toxic effects in the lung, spleen, and heart. These mice exhibited dose-dependent elevation of blood urea nitrogen, an indicator of kidney damage. Direct application of snPt1 to *in vitro* cultures of renal cells induced significant cytotoxicity. In mice administered for 4 weeks with twice-weekly intraperitoneal snPt1, histological analysis of the kidney revealed urinary casts, tubular atrophy, and inflammatory cell accumulation. Notably, these toxic effects were not observed in mice injected with 8-nm platinum particles, either by single- or multiple-dose administration. Our findings suggest that exposure to platinum particles of less than 1 nm in size may induce nephrotoxicity and disrupt some kidney functions. However, this toxicity may be reduced by increasing the nanoparticle size.

## Background

Nanomaterials have been developed and used as innovative materials in a wide range of industrial fields, including electronics, medicine, food, clothing, and cosmetics; these reagents are expected to provide significant benefits to humans. Nanomaterials are defined as substances that have at least one dimension size below 100 nm. The reduced size provides novel physicochemical properties, including increased thermal electrical conductivity, durability, and strength [1-3]. Although these characteristics may yield improved performance and novel functions, several reports have suggested that various types of nanomaterials, such as carbon nanotubes, titanium dioxide, fullerenes, quantum dots, and silica, exhibit harmful biological effects [4-12]. Additionally, some reports have shown that the characteristics of nanoparticles (e.g., size and surface features) can affect their biological and pathological actions [10,13-16]. Therefore, evaluation of the potential health risks attributable to nanomaterials is indispensable for the safe handling and use of these materials. However, little information is available regarding the safety evaluation of materials less than 1 nm in size.

Platinum nanoparticles have been utilized in a number of manufacturing applications, including catalysis, cosmetics manufacturing, and the processing of dietary supplements. As products using platinum nanoparticles become more familiar in our daily lives, the chances of exposure to platinum nanoparticles are increasing, as are concerns about unanticipated harmful biological effects of these materials [17,18]. In fact, there are some reports that platinum nanoparticles can induce inflammation in mice or impair the integrity of DNA [19,20]. On the other hand, platinum nanoparticles have anti-oxidant activity and inhibit pulmonary inflammation (e.g., as caused by exposure to cigarette smoke) [21-23]. These reports indicate that the biological effects of platinum nanoparticles remain poorly defined; the biological safety of sub-nanosized platinum particles (those of less than 1 nm in size; snPt1) remains unknown. Recently, we reported that snPt1 can induce hepatotoxicity [24]. However, the biological effects of snPt1 on other organs remain unclear. In this study, we evaluated the effect of snPt1 on tissues after single- and multi-dose administration in mice. In addition, we investigated the relationship between platinum particle size and biological response by also testing platinum particles of 8 nm in size (snPt8).

## Methods

### Platinum particles

Platinum particles with nominal mean diameters of less than 1 nm (snPt1) and 8 nm (snPt8) were purchased from Polytech & Net GmbH (Rostock, Germany). The particle sizes were confirmed using a Zetasizer Nano-ZS (Malvern Instruments, Malvern, UK). The particles were stocked as 5 mg/ml aqueous suspensions. The stock solutions were suspended using a vortex mixer before use. Other reagents used in this study were of research grade.

### Animals

BALB/c and C57BL/6 male mice were obtained from Shimizu Laboratory Supplies Co., Ltd. (Kyoto, Japan) and were housed in an environmentally controlled room at 23°C ± 1.5°C with a 12-h light/12-h dark cycle. Mice had *ad libitum* access to water and commercial chow (Type MF, Oriental Yeast, Tokyo, Japan). BALB/c mice were injected intravenously with snPt1 or snPt8 at 5 to 20 mg/kg body weight. C57BL/6 mice were injected intraperitoneally with snPt1 or snPt8 at 10 mg/kg body weight, or with an equivalent volume of vehicle (water). At 24 h after the injection of the vehicle or test article, the kidney and liver were collected. For testing the chronic effects of platinum particles, C57BL/6 mice were injected intraperitoneally with snPt1 or snPt8 at 10 mg/kg body weight, or with an equivalent volume of vehicle (water). Intraperitoneal doses were administered as twice-weekly injections for 4 weeks. At 72 h after the last injection of vehicle or test article, the kidney and liver were collected. All experimental protocols conformed to the ethical guidelines of the Graduate School of Pharmaceutical Sciences at Osaka University.

### Histological analysis

For animals dosed intravenously with snPt1 or snPt8, the kidney, spleen, lung, heart, and liver were removed at 24 h post-injection and fixed with 4% paraformaldehyde. For animals dosed intraperitoneally with snPt1 or snPt8, the kidney and liver were removed at 24 h (for single administration) or 72 h (for multiple administration) post-injection and fixed with 4% paraformaldehyde. Thin tissue sections were stained with hematoxylin and eosin for histological observation.

### Biochemical assay

Serum blood urea nitrogen (BUN) was measured using a commercially available colorimetric assay kit (Wako Pure Chemical, Osaka, Japan) according to the manufacturer’s protocol. In brief, collected serum (10 μl) was combined with 1 ml color A reagent (including urease) and incubated at 37°C for 15 min. Following the addition of 1 ml Color B reagent, the samples were incubated at 37°C for 10 min. Absorbance of samples was measured at a wavelength of 570 nm.

### Statistical analysis

Data are presented as mean ± SEM. Statistical analysis was performed by Student’s *t* test. *P* < 0.05 was considered significant.

## Results and discussion

**Figure 1:**
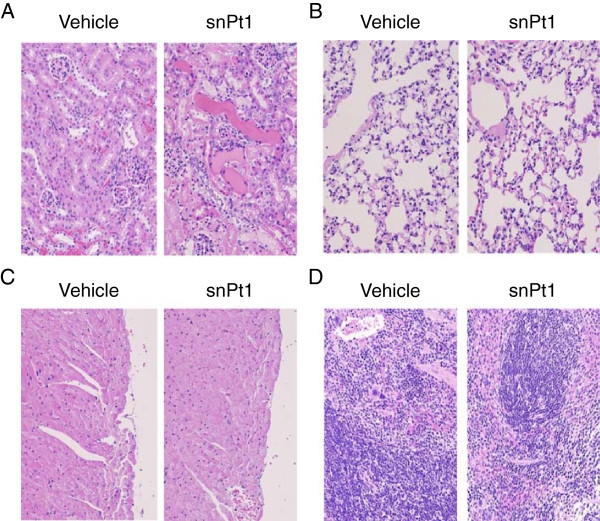
**Histological analysis of the organs in snPt1-treated mice.** Vehicle (water) or snPt1 (15 mg/kg) was administered intravenously to mice. At 24 h after administration, the kidney **(A)**, lung **(B)**, heart **(C)**, and spleen **(D)** were collected and fixed with 4% paraformaldehyde. Tissue sections were stained with hematoxylin and eosin and observed microscopically.

**Figure 2:**
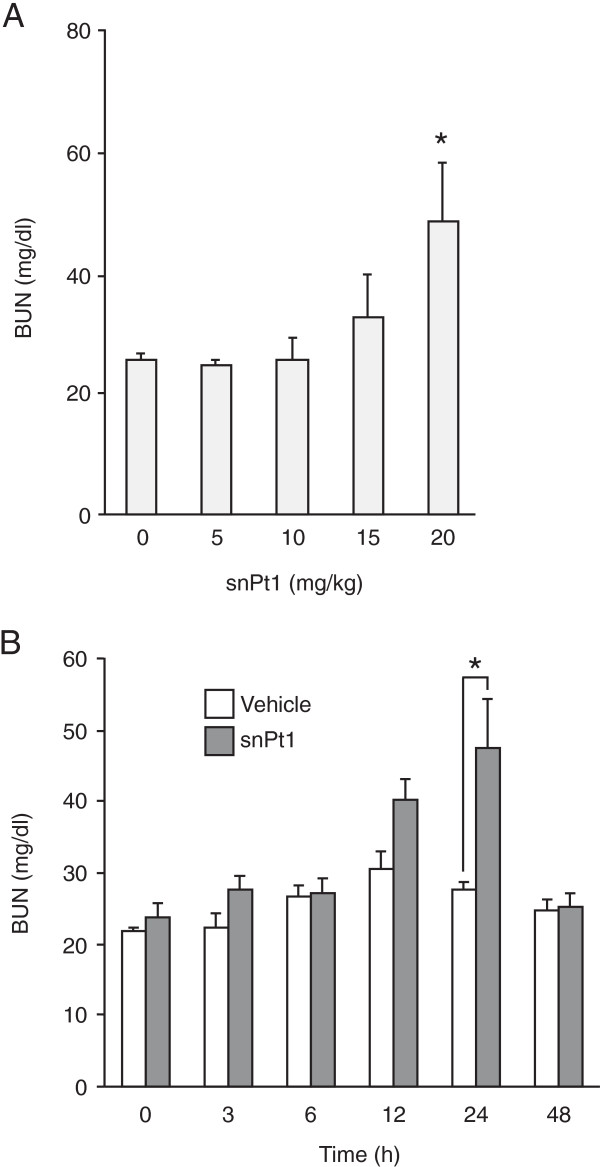
**Biochemical analysis in snPt1-treated mice. (A)** Dose dependency of snPt1-induced kidney injury. snPt1 was administrated intravenously at 5, 10, 15, or 20 mg/kg. At 24 h after administration, blood was recovered, and serum was collected and used for measurement of BUN, as described in the ‘Methods’ section. Data are mean ± SEM (*n* = 6 to 10). Single asterisk (*) connotes a significant difference when compared with the vehicle-treated group (*P* < 0.05). **(B)** Time-dependent changes in a biological marker of kidney injury. snPt1 was administered intravenously to mice at 20 mg/kg. Blood was recovered at 0, 3, 6, 12, 24, and 48 h after administration. Serum was collected and used for measurement of BUN, as described in the ‘Methods’ section. Data are mean ± SEM (*n* = 8 to 10). Single asterisk (*) connotes significant difference when compared with the vehicle-treated group (*P* < 0.05).

To investigate acute biological effects of snPt1, we administered 15 mg/kg of snPt1 to BALB/c mice by intravenous injection and performed histological analysis in the kidney, lung, heart, liver, and spleen at 24 h post-injection. As shown in Figure 1, necrosis of tubular epithelial cells and urinary casts were observed in the kidney by hematoxylin-eosin staining, whereas no apparent tissue abnormality was observed in the lung, heart, and spleen. Consistent with previous results [24], the liver showed vacuole degeneration after the administration of snPt1 (data not shown). These observations indicate that snPt1 induced acute tissue injury in the kidney and liver following intravenous administration. Next, we examined a serum biochemical marker of kidney function, BUN, to confirm the kidney tissue toxicity. Consistent with the histological analysis, intravenous dosing with snPt1 elevated serum BUN level at doses over 15 mg/kg (Figure 2A). The serum BUN level increased 24 h later and returned to normal level after 48 h (Figure 2B). When we directly added snPt1 at concentrations of 10, 20, 40, and 60 μg/ml to *in vitro* cultures of Madin-Darby canine kidney (MDCK) cells, severe cytotoxicity was observed in a dose-dependent manner (Additional file 1: Figure S1). These results indicate that snPt1 (at doses of greater than or equal to 15 mg/kg) induced toxicity in both the kidney and liver, but not in the lung, heart, or spleen, after a single intravenous administration.

**Figure 3:**
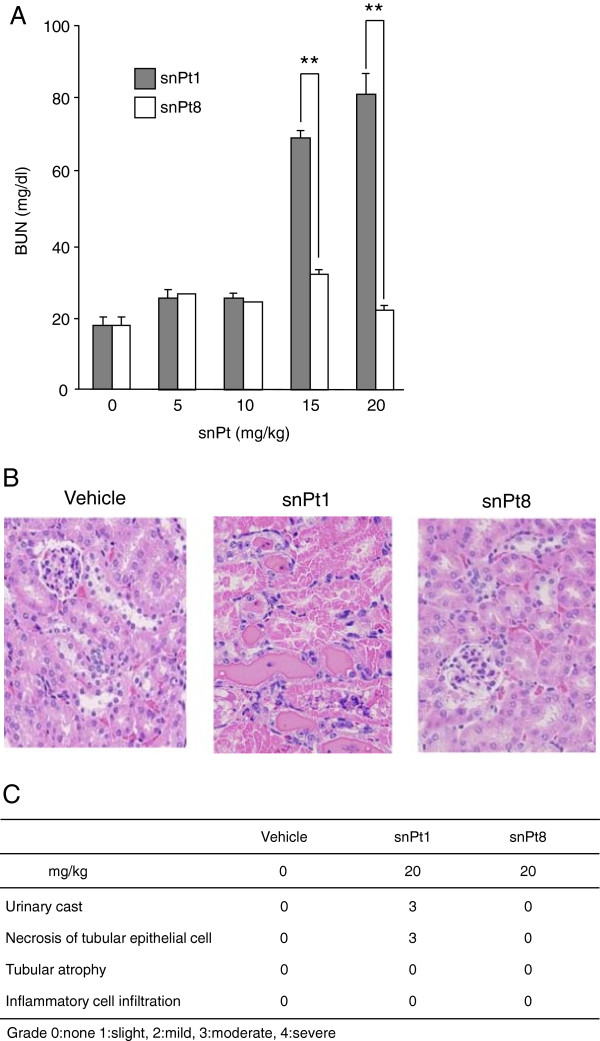
**Effect of particle size of platinum on kidney injury. (A)** snPt1 or snPt8 was injected intravenously into mice at the indicated doses. Blood was recovered at 24 h after injection. Serum BUN levels were measured. Data are mean ± SEM (*n* = 5). Double asterisk (**) connotes significant difference between the snPt1- and snPt8-treated groups (*P* < 0.01). **(B)** Histological analysis of kidney tissues in acute snPt1- or snPt8-treated mice. Vehicle or test article (snPt1 or snPt8 at 20 mg/kg) was administered intravenously to mice as a single dose. At 24 h after administration, the kidneys were collected and fixed with 4% paraformaldehyde. Tissue sections were stained with hematoxylin and eosin and observed under a microscope. **(C)** Acute kidney injury score in mice treated with vehicle, snPt1, or snPt8. Grade 0: none, 1: slight, 2: mild, 3: moderate, 4: severe.

Previously, we and other groups reported that the biological effects of nanoparticles differed with material size [10,11,25,26]. Therefore, we examined whether platinum particles with a diameter of 8 nm (snPt8) and snPt1 produce different effects in kidney. As shown in Figure 3A, snPt1 administration resulted in dose-dependent increases in serum BUN levels, whereas snPt8 (at the same dose levels) did not. Histological analysis showed that intravenous administration (at 20 mg/kg) of snPt1, but not that of snPt8, induced renal injury (Figure 3B,C). These tissue injuries also were observed following the injection in C57BL/6 mice (data not shown), demonstrating that the toxicity was not mouse strain-specific. Furthermore, renal cytotoxicity was not observed in snPt8-treated MDCK cells (Additional file 1: Figure S1), confirming the size dependence of the nanoparticle renal cytotoxicity. The hepatotoxicity of the platinum particles also was reduced by altering particle size [24]. These findings indicate that the snPt1-induced nephrotoxicity is not observed following treatment with the same dose level of snPt8.

**Figure 4:**
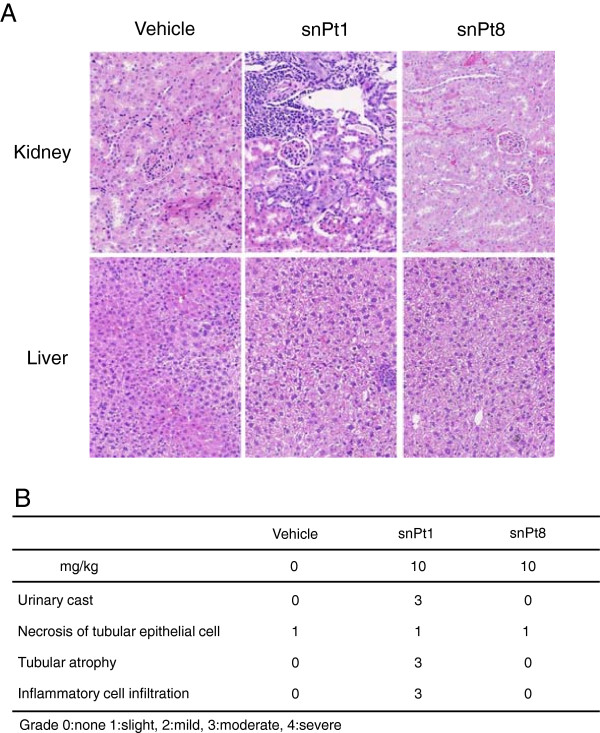
**Histological analysis of kidney tissues in multi-dose snPt1- or snPt8-treated mice. (A)** Vehicle or test article (snPt1 or snPt8 at 10 mg/kg) was administered intraperitoneally to mice as twice-weekly doses for 4 weeks. At 72 h after last administration, the kidney and liver were collected and fixed with 4% paraformaldehyde. Tissue sections were stained with hematoxylin and eosin and observed under a microscope. **(B)** Chronic kidney injury scores in mice treated with vehicle, snPt1, or snPt8. Grade 0: none, 1: slight, 2: mild, 3: moderate, 4: severe.

Finally, we used histological analysis to investigate the effects on C57BL/6 mice of chronic exposure to snPt1 and snPt8. snPt1 and snPt8 (both at 10 mg/kg) were injected intraperitoneally into mice twice per week for 4 weeks; repeat administration via the tail vein was precluded due to tissue necrosis of the mouse tail upon multiple intravenous administrations. In the multiple intraperitoneal administrations, necrosis at the injection site was not observed. Single intraperitoneal administration of 10 mg/kg snPt1 (but not that of snPt8) induced necrosis of tubular epithelial cells and urinary casts in the kidney, similar to the results seen with intravenous administration (Additional file 2: Figure S2A,B). Chronic intraperitoneal administration of snPt1 at 10 mg/kg induced urinary casts, tubular atrophy, and inflammatory cell accumulation in the kidney, whereas the liver did not show tissue injury (Figure 4A,B). On the other hand, chronic exposure to snPt8 (at the same dose level) did not show apparent histological effects in the kidney (Figure 4A,B). These findings suggest that chronic exposure to 10 mg/kg snPt1, but not to snPt8, induced severe kidney injury. Notably, this chronic exposure to snPt1 induced additional (cumulative) kidney injury beyond that seen with acute exposure.

Following exposure, nanoparticles are transported into the blood and reach the systemic circulation, from which the nanoparticles distribute and accumulate in several organs such as the lung, liver, spleen, kidneys, brain, and heart [27-30]. Because the kidney is able to remove molecules from the circulation, renal excretion is an expected route for elimination of nanoparticles. In fact, functionalized single-wall carbon nanotubes (SWCNT), following injection into mice, are rapidly excreted by the kidney [31]. The hepatobiliary system also is an important route for the elimination of foreign substances and particles [32]. Because these organs play pivotal roles in eliminating foreign substances, various nanomaterials are accumulated there and lead to tissue injury. As one example, our previous work showed that snPt1-treated mice exhibited acute hepatotoxicity [24]. In the present study, we investigated the biological effects of snPt1 after intravenous or intraperitoneal administration in mice and demonstrated that snPt1 induced nephrotoxicity and impaired renal function, as evidenced by BUN levels. In contrast, we could not find apparent toxic effects on the heart, lung, or spleen after the single intravenous administration of snPt1, although the disposition of these nanoparticles will need to be assessed further.

The underlying mechanism of snPt1-induced tissue injury still remains unclear. Cisplatin, which is a platinating agent used as part of the anti-cancer regimen for various types of cancers [33,34], exerts its antitumor activity by binding preferentially to the nucleophilic positions on guanine and adenine of DNA, resulting in the formation of intra- and inter-strand crosslinks. Eventually, the crosslinks lead to DNA-strand breaks and killing of cancer cells [35]. However, cisplatin usage is limited due to nephrotoxicity, leading to lesions in the epithelial tubules [36,37]. Cisplatin also causes toxicity in the liver and blood [38]. These observations suggest that the toxic effects of cisplatin resemble those of snPt1. A previous study reported that platinum nanoparticles entered human lung fibroblasts (cell line IMR-90) and human glioblastoma cells (U251) and induced cytotoxicity thorough intracellular reactive oxygen species (ROS) production and DNA damage following p53 activation and upregulation of p21, which leads to growth arrest and apoptosis [39]. Our observation of snPt1-induced cytotoxicity in cell culture suggests that snPt1 may be internalized by renal cells, with concomitant induction of ROS production or DNA damage. However, alternative toxic effects (such as cytotoxicity of inflammatory cytokines on renal cells by accumulation of inflammatory cells in the kidney) might emerge during chronic exposure to snPt1.

At equivalent dose levels, platinum particles of 8 nm in size did not induce apparent toxic effects in renal tissues by acute or chronic administration. This result suggests that selection of specific size ranges for the platinum particles might overcome the undesirable side effects. Current studies have shown that organic cation transporter 2 (OCT2) is highly expressed in kidney and plays an important role in the nephrotoxicity of cisplatin [40,41]. Identification of the snPt1 transporter may help to clarify the mechanism of snPt1-induced nephrotoxicity.

## Conclusions

In the present study, we investigated the biological safety of platinum nanoparticles in mice and found that platinum particles of less than 1 nm induced kidney injury, although the injurious effects were reduced by increasing the nanoparticle size. For future nanoparticle applications, it will be critical to further understand the bioactivity and kinetics of materials less than 1 nm in size. Accumulation of toxicity profiles will aid in the creation of the safe and efficacious nanomaterials and contribute to the advancement of the field.

## Abbreviations

snPt1: platinum particles less than 1 nm in size; snPt8: platinum particles of 8 nm in size; BUN: blood urea nitrogen; MDCK: Madin-Darby canine kidney; ROS: reactive oxygen species.

## Competing interests

The authors declare that they have no competing interests.

## Authors’ contributions

AW, MK, and KY designed this study. YY (Yoshioka) and YT prepared samples. YY (Yamagishi), YH, and XL performed the experiments. AW and KY wrote this manuscript. All authors read and approved the final manuscript.

## Supplementary Material

Additional file 1: Figure S1 Cytotoxicity of snPt1 in renal cells. MDCK cells were treated with vehicle, snPt1, or snPt8 at 0, 10, 20, 40, or 60 μg/ml. After 24 h exposure, morphology of the cells was photographed. Higher magnification images are shown in the insets. Click here for file

Additional file 2: Figure S2 (A) Histological analysis of kidney tissues in intraperitoneally administered mice. Vehicle or test article (snPt1 or snPt8 at 10 mg/kg) was administered intraperitoneally to mice as a single dose. At 24 h after administration, kidneys were collected and fixed with 4% paraformaldehyde. Tissue sections were stained with hematoxylin and eosin and observed under a microscope. (B) Acute kidney injury score in mice treated intraperitoneally with vehicle, snPt1, or snPt8. Grade 0: none, 1: slight, 2: mild, 3: moderate, 4: severe. Click here for file
