# Supplementary figures and images for: Acute and chronic nephrotoxicity of platinum nanoparticles in mice
Source: Nanoscale Res Lett. 2013 Sep 23;8(1):395. doi: 10.1186/1556-276X-8-395 (PMC3849727; doi:10.1186/1556-276X-8-395)

## Slide 1
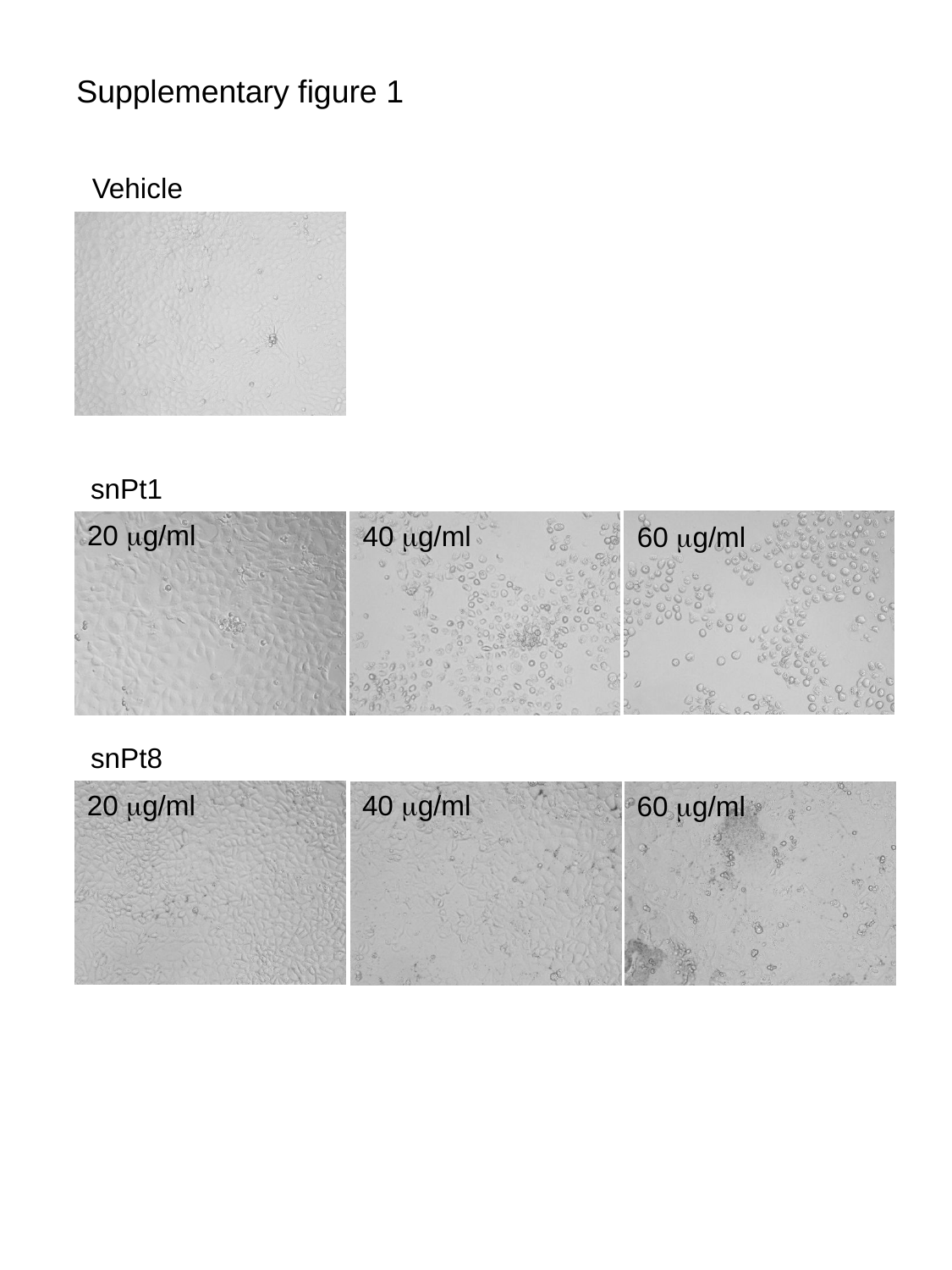

Supplementary figure 1
Vehicle
snPt1
20 μg/ml
40 μg/ml
60 μg/ml
snPt8
20 μg/ml
40 μg/ml
60 μg/ml

Supplement: Additional file 1: Figure S1 — Cytotoxicity of snPt1 in renal cells. MDCK cells were treated with vehicle, snPt1, or snPt8 at 0, 10, 20, 40, or 60 μg/ml. After 24 h exposure, morphology of the cells was photographed. Higher magnification images are shown in the insets. [file 1556-276X-8-395-S1.ppt]
