# Supplementary material for: Acute and chronic nephrotoxicity of platinum nanoparticles in mice
Source: Nanoscale Res Lett. 2013 Sep 23;8(1):395. doi: 10.1186/1556-276X-8-395 (PMC3849727; doi:10.1186/1556-276X-8-395)
Supplement: Additional file 2: Figure S2 — (A) Histological analysis of kidney tissues in intraperitoneally administered mice. Vehicle or test article (snPt1 or snPt8 at 10 mg/kg) was administered intraperitoneally to mice as a single dose. At 24 h after administration, kidneys were collected and fixed with 4% paraformaldehyde. Tissue sections were stained with hematoxylin and eosin and observed under a microscope. (B) Acute kidney injury score in mice treated intraperitoneally with vehicle, snPt1, or snPt8. Grade 0: none, 1: slight, 2: mild, 3: moderate, 4: severe. [file 1556-276X-8-395-S2.ppt]

## Slide 1
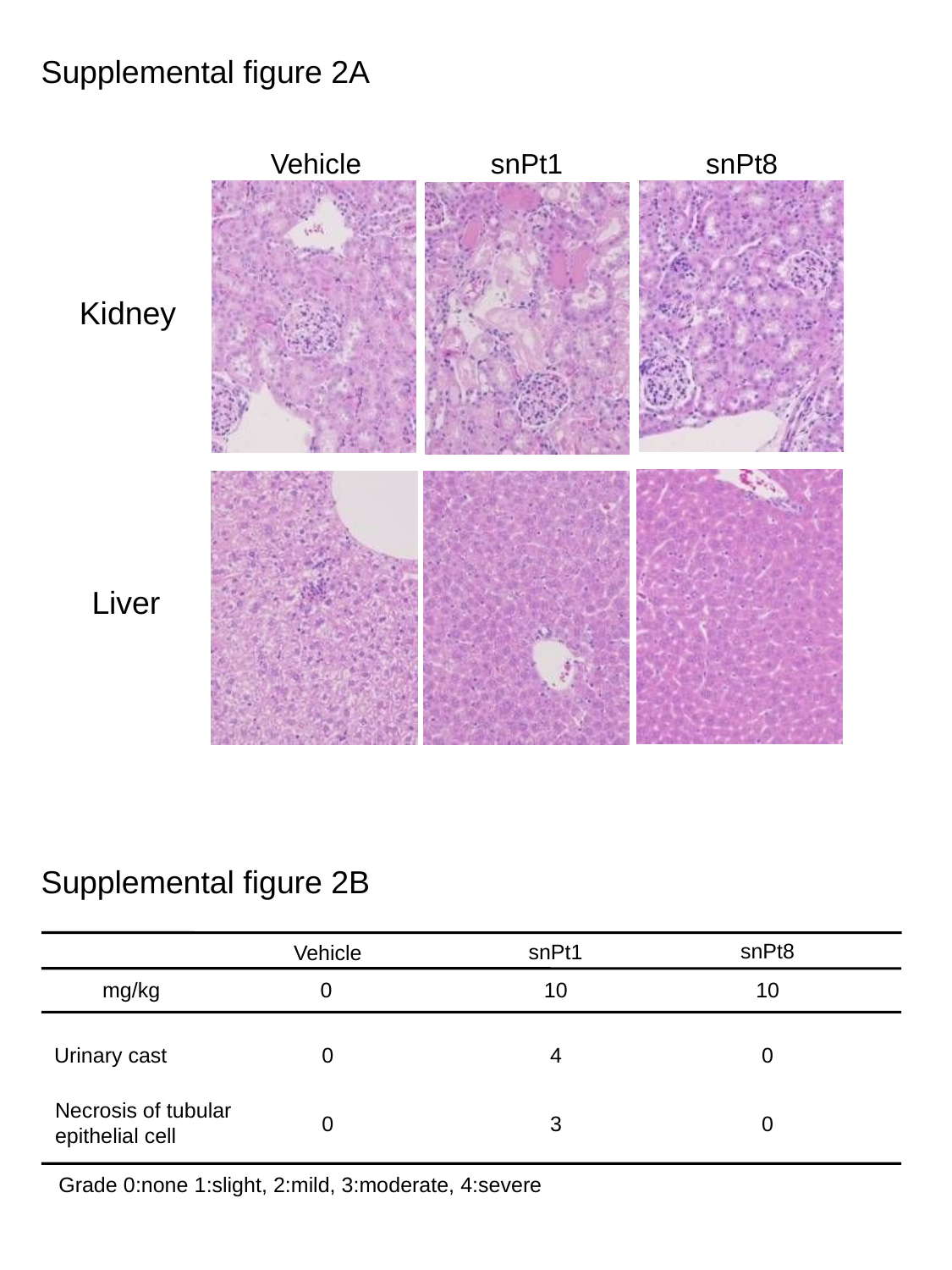

Supplemental figure 2A
Vehicle
snPt1
snPt8
Kidney
Liver
Supplemental figure 2B
snPt8
snPt1
Vehicle
mg/kg
0
10
10
Urinary cast
0
4
0
Necrosis of tubular
epithelial cell
0
3
0
Grade 0:none 1:slight, 2:mild, 3:moderate, 4:severe
